# A deep neural network prediction method for diabetes based on Kendall’s correlation coefficient and attention mechanism

**DOI:** 10.1371/journal.pone.0306090

**Published:** 2024-07-02

**Authors:** Xiaobo Qi, Yachen Lu, Ying Shi, Hui Qi, Lifang Ren

**Affiliations:** 1 School of Computer Science and Technology, Taiyuan Normal University, Jinzhong, Shanxi, China; 2 Shanxi Key Laboratory of Intelligent Optimization Computing and Blockchain Technology, Jinzhong, Shanxi, China; 3 School of Computer and Information Technology, Shanxi University, Taiyuan, Shanxi, China; 4 School of information, Shanxi University of Finance and Economics, Taiyuan, PR China; Abdul Wali Khan University Mardan, PAKISTAN

## Abstract

Diabetes is a chronic disease, which is characterized by abnormally high blood sugar levels. It may affect various organs and tissues, and even lead to life-threatening complications. Accurate prediction of diabetes can significantly reduce its incidence. However, the current prediction methods struggle to accurately capture the essential characteristics of nonlinear data, and the black-box nature of these methods hampers its clinical application. To address these challenges, we propose KCCAM_DNN, a diabetes prediction method that integrates Kendall’s correlation coefficient and an attention mechanism within a deep neural network. In the KCCAM_DNN, Kendall’s correlation coefficient is initially employed for feature selection, which effectively filters out key features influencing diabetes prediction. For missing values in the data, polynomial regression is utilized for imputation, ensuring data completeness. Subsequently, we construct a deep neural network (KCCAM_DNN) based on the self-attention mechanism, which assigns greater weight to crucial features affecting diabetes and enhances the model’s predictive performance. Finally, we employ the SHAP model to analyze the impact of each feature on diabetes prediction, augmenting the model’s interpretability. Experimental results show that KCCAM_DNN exhibits superior performance on both PIMA Indian and LMCH diabetes datasets, achieving test accuracies of 99.090% and 99.333%, respectively, approximately 2% higher than the best existing method. These results suggest that KCCAM_DNN is proficient in diabetes prediction, providing a foundation for informed decision-making in the diagnosis and prevention of diabetes.

## Introduction

Diabetes is a chronic metabolic disease characterized by elevated blood glucose levels due to insufficient insulin secretion or impaired insulin action. In severe cases, it can lead to various complications, including cardiovascular disease, kidney damage, neuropathy, and retinopathy [1]. According to statistics from the International Diabetes Federation(IDF): approximately 537 million adults (ages 20-79) worldwide had diabetes in 2021. This number is projected to increase to 643 million by 2030 and further to 783 million by 2045 [2]. The Children’s Diabetes Association notes a concerning trend of diabetes affecting a younger demographic, with around 1.1 million people under the age of 20 worldwide diagnosed with type 1 diabetes [3]. The escalating incidence of diabetes poses a significant challenge to global public health. Given the absence of a cure for diabetes, proactive prevention becomes crucial. Accurate prediction of diabetes plays a vital role in providing technical support for disease prevention efforts.

In recent years, extensive research has been conducted on predicting diabetes, utilizing various machine learning algorithms for diabetes data. Traditional machine learning models, including Random Forest (RF) [4], Support Vector Machine (SVM) [5], Logistic Regression (LR) [6], and k-nearest Neighbor(KNN) [7], and others, have been employed to build predictive models aimed at assisting physicians in diagnostic decision-making. However, these methods are grounded in linear models and lack the capability to effectively model complex nonlinear data. Prediction based on traditional models often falls short of meeting the performance requirements for clinical applications, with accuracy levels below 90%.

It’s notable that some scholars have ventured into using deep learning methods for diabetes prediction research [8]. Deep learning proves advantageous in handling complex nonlinear data, as it has the capability to automatically learn feature representations, consequently improving prediction accuracy [9]. Deep learning models can be highly sensitive to the representation of input data and often require careful consideration of feature selection to optimize performance. Another critical concern is that deep learning models are frequently perceived as black boxes, given their intricate internal structures and decision-making processes, making it challenging to explain and understand how predictions are derived.

To enhance both the accuracy and interpretability of diabetes prediction, we propose KCCAM_DNN, a high-performance deep neural network prediction method for diabetes. KCCAM_DNN integrates Kendall’s correlation coefficient and an attention mechanism, providing a robust solution for early disease detection and prevention. By effectively controlling disease progression, this approach aims to reduce the incidence of diabetes. We conducted experiments on PIMA Indian and LMCH diabetes datasets, achieving remarkable results. Specifically, the test accuracies reached 99.090% and 99.333% respectively, showcasing an improvement of approximately 2% compared to the best available method. The key contributions and findings of this work are summarized as follows:

1. **Feature Analysis and Selection**: The method employs Kendall’s Correlation Coefficient(KCC) to analysis and select features effectively, overcoming the challenge of identifying relevant features in a large pool of variables. This helps to reduce the dimensionality of the feature space and eliminates redundant or irrelevant features, improving the efficiency of the prediction model. Apply polynomial regression(PR) to interpolate missing values, enhancing data completeness.
2. **Model Construction with Self-Attention Mechanism**: Introduce the self-attention mechanism to construct the KCCAM_DNN model based on a deep neural network. The incorporation of self-attention mechanism in the hidden layers of the deep neural network (DNN) enables the model to learn the associations and importance between features. This attention mechanism helps the model focus on relevant features, particularly those crucial for diabetes prediction. By emphasizing important features during training, the model can improve its predictive performance. Fine-tune model hyperparameters through grid search and repeat stratified k-fold cross-validation to improve performance. Conduct comparative experiments, demonstrating the superiority of the model across various prediction metrics.
3. **Dataset Relabeling for Prediabetes Prediction**: Relabel the PIMA Indian dataset to enable the prediction of prediabetes classes. Provide a more comprehensive clinical diagnosis of diabetes.
4. **Interpretability Enhancement**: Adopt the SHAP (SHapley Additive exPlanations) model to analyze the main features influencing diabetes prediction. The incorporation of the SHAP model enhances the interpretability of the KCCAM_DNN model because it offers insights on the importance of individual features in predicting diabetes. As a result, researchers and clinicians are able to understand the underlying factors contributed by each feature. This transparency is crucial for gaining insights into the prediction process and for building trust in the model’s results.

## Related work

In recent years, propelled by the rapid progress of science and technology, researchers have undertaken extensive and in-depth explorations in the realm of diabetes prediction. The primary emphasis within this field has centered on methodologies grounded in both machine learning and deep learning.

Kangra et al. [10] used machine learning algorithms for prediction of diabetes mellitus, validated on Pima Indian Diabetes Datasets (PIDD) dataset. The results show that SVM works better and has 74% accuracy. However, the hybrid model proposed in the article has no relevant experiments and lacks comparability. Sai M J et al. [11] proposed machine learning ensemble algorithm for predicting diabetes. The algorithm ensembles KNN, Naive Bayes, Random Forest, Adaboost and Light Gradient Boosting Machine. KNN, Adaboost and LightGBM together achieved 90.76% accuracy and successfully solved the class imbalance problem of the underlying dataset. Edeh et al. [12] used four machine learning classification algorithms to predict diabetes, namely supervised learning algorithms (Random forest, SVM and Naive Bayes) and unsupervised learning algorithm (k-means). The results obtained from Pima Indian database showed that SVM algorithm has the highest accuracy of 83.1%. However, the model exhibited significant memory and computation time requirements, particularly when confronted with high-dimensional data. In a separate study, Rupapara et al. [13] presented an approach to classify diabetic and normal individuals using integrated machine learning models. Several experiments were conducted on the PIMA dataset, employing Principal Component Analysis (PCA) and chi-square (Chi-2) features, resulting in an accuracy of 85%. Nonetheless, the effectiveness of this method on unstructured datasets remains unknown.

In summary, machine learning methods heavily depend on the quality and quantity of data, posing challenges when dealing with missing and noisy data. Issues such as overfitting may arise when the model is overly complex or the training data is insufficient, leading to suboptimal performance on new data [14]. Deep learning methods, on the other hand, exhibit a capacity to effectively capture intricate relationships within data, resulting in enhanced model performance. These methods excel in processing unstructured data and demonstrate greater adaptability to complex data types compared to traditional machine learning approaches.

Chollette et al. [15] introduced a 2GDNN (two-growth deep neural network) diabetes prediction model, utilizing a deep neural network as the foundational framework. The model was validated on the PIMA and LMCH datasets, achieving test accuracies of 97.248% and 97.333%, respectively. However, it’s worth noting that the 2GDNN prediction model employs the Spearman correlation coefficient for feature selection. The stability of Spearman correlation coefficient results may be compromised when dealing with small sample data. Additionally, 2GDNN is characterized as a black-box model, lacking interpretability. In a separate study, Khanam and Foo [16] utilized Artificial Neural Network (ANN) for diabetes prediction, attaining an accuracy of 88.6% on the PIMA dataset. Jaloli et al. [17] designed a deep neural network model based on CNN-LSTM, combining a stack of Convolutional Neural Network (CNN) and Long Short-Term Memory (LSTM) units to predict blood glucose levels for 30-, 60-, and 90-minute prediction horizons (PH). The study used the Replace-BG and DIAdvisor datasets, achieving a maximum detection accuracy of 94.71%. However, it is important to note that dealing with large-scale datasets or long time series data may especially result in high time and resource consumption for training and inference.

In summary, despite the advancements in existing algorithms for diabetes prediction, several challenges persist:

1) Diabetes data is inherently nonlinear, with complex relationships among variables. However, most of the current diabetes predictions models employ linear data processing methods for feature selection, limiting their effectiveness.
2) Existing deep learning models treat all feature information equally and may not effectively prioritize important features.
3) Deep models are typically complex black-box models that are challenging to interpret and lacking in model interpretability.

To address these issues, we propose KCCAM_DNN. The Kendall’s correlation coefficient utilized in feature selection effectively sifts through the primary features impacting diabetes prediction, thereby eliminating redundancy. Adding self-attention mechanism into the hidden layers before and after the output layer, helps the deep neural network model understand the association and significance of features, so that it can give priority to the important features affecting diabetes. Therefore, these enhancements enhance the model’s ability to identify key features and improve its predictive performance. Combined with the SHAP model, KCCAM_DNN can explain the contribution of key features to diabetes prediction. This helps us understand which features play a key role in prediction and provides a further explanation of the model. Feature selection improves classifier performance by 1.334%, 7.5%, and 0.915% for the SVM, RF, and KCCAM_DNN models, respectively. Compared to the existing best available methods, the addition of the self-attention mechanism improved test accuracy by 2%. It aims to enhance the accuracy of diabetes prediction by addressing the nonlinear nature of diabetes data and prioritizing important features. The introduction of KCCAM_DNN facilitates precise diabetes prediction, offering valuable technical support for medical diagnosis and prevention of diabetes.

## Methods

**Fig 1:**
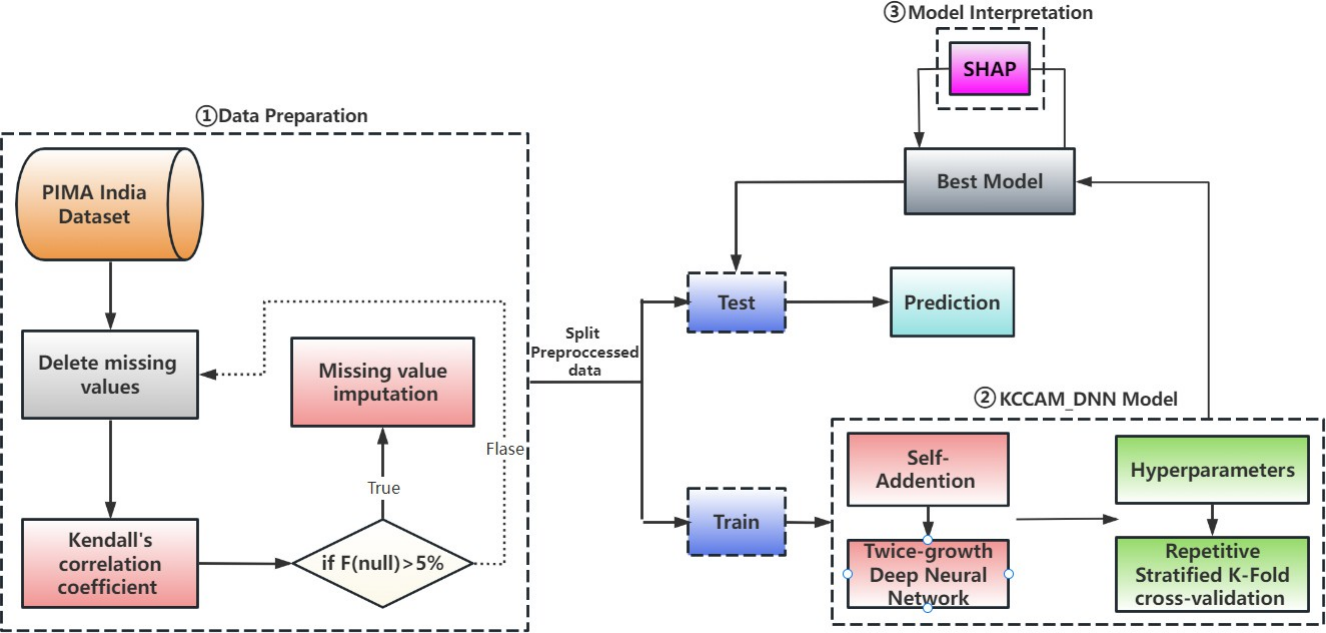
Workflow of KCCAM_DNN.

KCCAM_DNN comprises three main modules(Fig 1):(i)Data preprocessing. Feature selection is performed using Kendall’s correlation coefficient, and polynomial regression is applied for imputation on the original data with missing values. (ii)Model construction. A DNN model based on the self-attention mechanism, named KCCAM_DNN, is designed and implemented. Model hyperparameters are fine-tuned through grid search and repeated stratified k-fold cross-validation. (iii)Model interpretation. The SHAP model is incorporated to analyze the main features influencing the prediction results of diabetes, enhancing the model’s interpretability.

### Data preprocessing

The prevention of diabetes is more crucial than treatment, making accurate prediction of prediabetic conditions a significant research focus. While much of the existing diabetes research primarily centers around predicting whether an individual has diabetes or not, for improved diagnosis and prevention, it is essential to classify prediabetic conditions. The LMCH diabetes dataset comprises three categories: N (Normal), P (Prediabetes), and Y (Diabetes), eliminating the need for relabeling. In the PIMA Indian dataset, there are two categories: 0 (Normal) and 1 (Diabetes). We relabeled the dataset based on blood glucose levels into normal, prediabetes, and diabetes. In the subsequent processing, we identified and preserved the key features in the data using feature selection methods. Afterwards, we processed the missing values with suitable interpolation methods. This significantly reduces redundant data and improves data quality and processing efficiency.

#### Feature selection

Feature selection is a critical step in data preprocessing. Not all attributes in a dataset are necessarily important features for prediction. Selecting the features most correlated with the target variable can enhance prediction accuracy and expedite both model training and inference speed. Given that the diabetes dataset is nonlinear, Kendall’s correlation is preferred over Spearman correlation in terms of robustness and efficiency [18]. Consequently, we opt for Kendall’s correlation coefficient for feature selection.

Kendall’s correlation coefficient is a nonparametric test employed to evaluate the degree of association between two variables. Nonparametric correlation tests do not depend on assumptions about the distribution of the data but are instead based on the ranking or ordering of the data and pairwise comparisons. The Kendall’s correlation coefficient is calculated as follows:

$$\begin{array}{c} \tau =\frac{N_{c}-N_{d}}{n/2(n-1)} \end{array}$$ (1)

Here, *τ* represents the Kendall’s correlation coefficient, *N*_*c*_ is the number of concordant pairs, *N*_*d*_ is the number of discordant pairs, and *n* is the total number of pairs.

Kendall’s correlation coefficient ranges between -1 and 1. A value of 1 indicates a perfect positive correlation between two variables, meaning that when one variable increases, the other variable increases proportionally. A value of -1 indicates a perfect negative correlation, where an increase in one variable corresponds to a proportional decrease in the other variable. When the coefficient approaches 0, it implies a lack of linear correlation or weak linear correlation between the two variables.

The research results presented in Table 1 illustrate a noteworthy correlation between glucose and insulin levels with the target variable. These two features are deemed crucial and will constitute a subset of the original PIMA Indian dataset. The identical feature selection algorithm was utilized for the LMCH dataset and can be applied effectively to any other dataset.

**Table 1:** The statistical significance of Kendall’s correlation coefficient values between predictor and outcome variables is crucial for feature selection.

Feature	Glucose	Insulin	DPF	Age	BMI	Pregnancies	SkinThickness	BloodPressure
Glucose	1(0.00***)	0.516(0.00***)	0.059(0.38)	0.334(0.00***)	0.147(0.03**)	0.255(0.00***)	0.184(0.01***)	0.162(0.02**)
Insulin	0.516(0.00***)	1(0.00***)	0.042(0.54)	0.324(0.00***)	0.237(0.00***)	0.19(0.01***)	0.241(0.00***)	0.126(0.07*)
DPF	0.059(0.39)	0.042(0.54)	1(0.00***)	0.071(0.31)	0.000(0.99)	-0.026(0.71)	0.081(0.24)	0.021(0.76)
Age	0.334(0.00***)	0.324(0.00***)	0.071(0.31)	1(0.00***)	0.191(0.01***)	0.452(0.00***)	0.181(0.01***)	0.178(0.01**)
BMI	0.147(0.03**)	0.237(0.00***)	0.000(0.99)	0.191(0.01***)	1(0.00***)	-0.015(0.84)	0.488(0.00***)	0.201(0.00***)
Pregnancies	0.255(0.00***)	0.19(0.01***)	-0.026(0.72)	0.452(0.00***)	-0.015(0.84)	1(0.00***)	0.021(0.77)	0.121(0.09*)
SkinThickness	0.184(0.01***)	0.241(0.00***)	0.081(0.24)	0.181(0.01***)	0.488(0.00***)	0.02(0.768)	1(0.00***)	0.1(0.15)
BloodPressure	0.162(0.02**)	0.126(0.07*)	0.021(0.76)	0.178(0.01**)	0.201(0.00***)	0.121(0.09*)	0.1(0.15)	1(0.00***)
Outcome	0.813(0.00***)	0.548(0.00***)	0.114(0.14)	0.322(0.00***)	0.195(0.01**)	0.273(0.00***)	0.244(0.00***)	0.193(0.02**)

***,**,* represent 1%,5%,and 10% significance levels.

DPF:DiabetesPedigreeFunction.

#### Missing value imputation

Applying missing value imputation is crucial when dealing with datasets containing a large number of missing values. Directly removing samples with missing values may reduce the dataset’s sample size, diminishing the reliability and accuracy of model predictions. To enhance model performance, imputing missing values is employed to obtain a complete dataset. In the PIMA Indian dataset, which comprises a substantial number of missing values (432 out of 768 data points), missing value imputation is implemented, while the LMCH dataset, lacking any missing values, does not undergo this process.

The common practice in diabetes research is to fill missing data with mean and median values. However, these methods may introduce data bias [19]. Multiple Imputations of Missing Values (MICE) [20] is an alternative technique that predicts missing values using existing feature data. However, MICE is not well-suited for nonlinear data [21]. In the context of nonlinear relationships, polynomial regression is a more appropriate approach for describing correlations between features, offering improved data fitting. Consequently, we employ polynomial regression for missing value imputation.

Polynomial regression (PR) extends linear regression to model nonlinear relationships. In polynomial regression, polynomial terms, such as powers of the independent variables, are incorporated into the regression model. A standard monomial polynomial regression model is represented as follows:

$$\begin{array}{c} Y=\beta_{0}+\beta_{1}X+\beta_{2}X^{2}+\ldots +\beta_{n}X^{n}+\epsilon \end{array}$$ (2)

where *Y* is the dependent variable, *X* is the independent variable, *β*_0_ is the bias term, *β*_1_, *β*_2_, *β*_3_,…,*β*_*n*_ are regression coefficients, and *X* the predictor variable and *X*, *X*^2^,…, *X*^*k*^ are additional variables created by raising *X* to various exponents.

**Fig 2:**
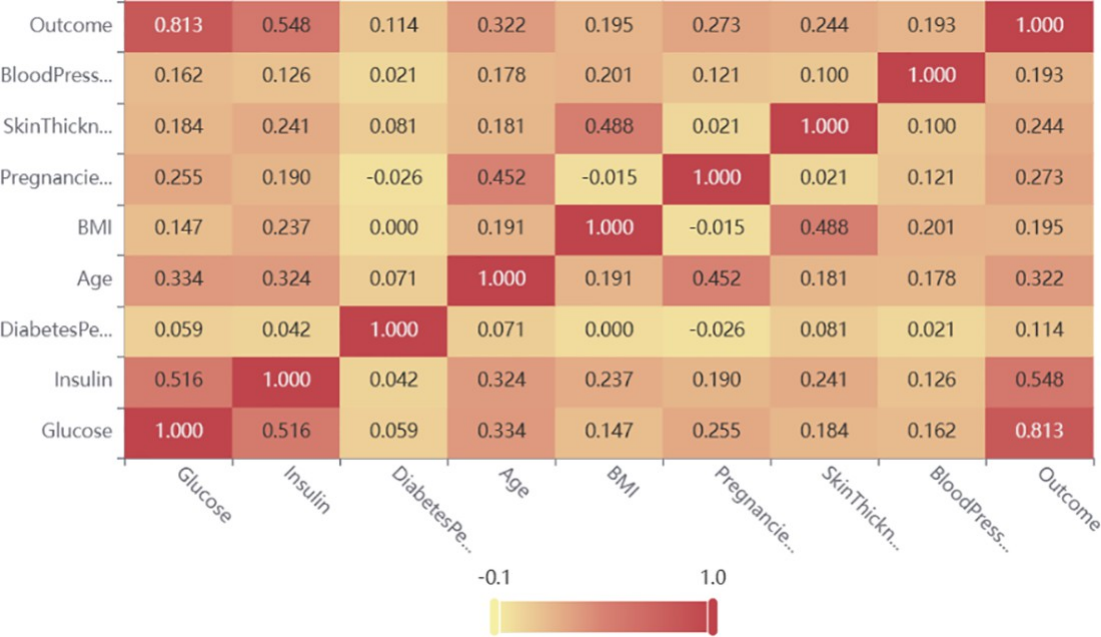
Correlation coefficient heat map. The map shows a significant relationship between Glucose and Insulin.

The primary objective of polynomial regression is to capture nonlinear relationships within the data by introducing power terms for the independent variables. For instance, introducing quadratic (*X*^2^) and cubic (*X*^3^) terms accommodates complex shapes like quadratic or cubic curves in the data [22]. If the order is too low, the model may fail to accurately fit the data. The process of performing polynomial regression interpolation involves the following steps:

(1) Identify all missing values in the subset of the PIMA Indian dataset after feature selection. Examine the percentage of missing values for features, setting the threshold at 5%. If the number of zero entries in the subset data is less than 5%, the entry is deleted; otherwise, polynomial regression (PR) is applied. For the PIMA Indian diabetes dataset, insulin has zero entries exceeding 5%.
(2) Referencing the correlation heat map in Fig 2, identify highly correlated variables. In this case, glucose and insulin are highly correlated, making glucose a predictive variable for filling in missing insulin values.
(3) Predicting missing values using polynomial regression. For each sample with missing values, input glucose to construct a polynomial regression model for prediction. Combine the resulting output with the non-missing values to form the final dataset.

### KCCAM_DNN

#### Model construction

The Deep Neural Network (DNN) is an artificial neural network model comprised of multiple layers of neurons, typically with multiple hidden layers. During the training process, the backpropagation algorithm is used to update the connection weights [23]. DNN has been widely applied to diabetes prediction [8]. However, DNN models have a large number of parameters and a complex structure, requiring a longer training time. DNN treats all feature information equally and may not effectively prioritize important features. There are also limitations in terms of interpretability, which is crucial for physicians and patients in diabetes research.

The traditional attention mechanism computes attention weights by sequentially calculating the relevance between different features, which leads to higher computational complexity. In contrast, self-attention mechanism performs parallel computations across all features, significantly improving computational efficiency. Moreover, it dynamically adjusts the weights based on the input context, allowing the model to better focus on relevant information, thereby enhancing the model’s perception of significant features [24]. This capability is valuable for predicting the health status and risk of diabetic patients.

**Fig 3:**
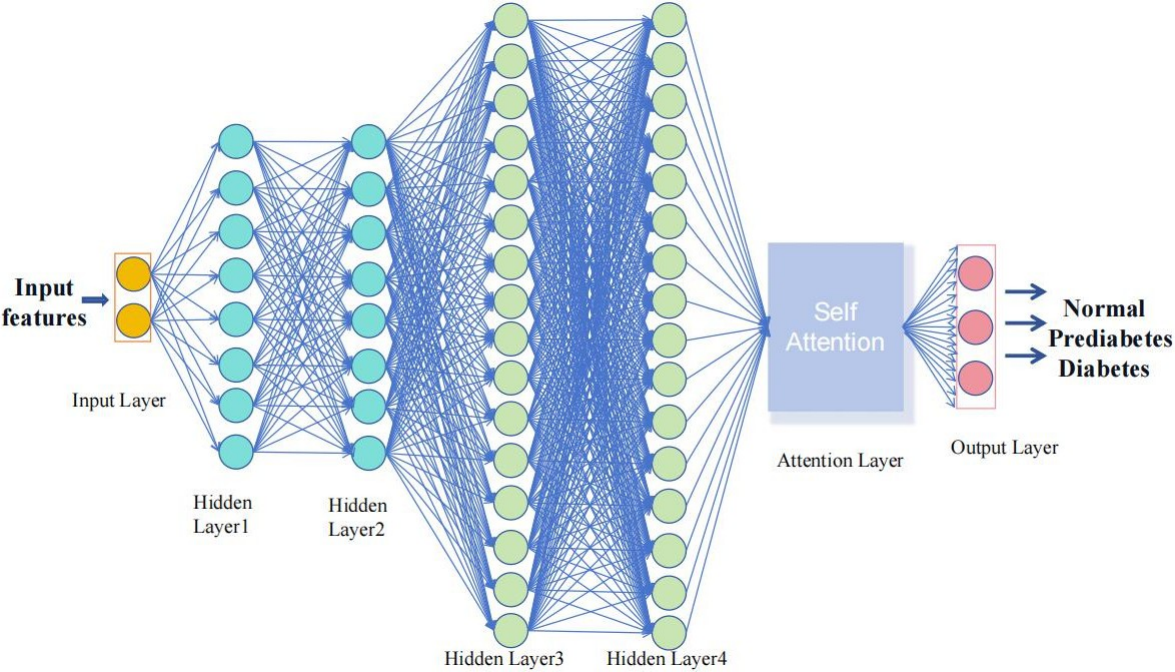
The architecture of the proposed deep neural network model based on Kendall’s correlation coefficient and attention mechanism (KCCAM_DNN) for diabetes prediction.

This work introduces KCCAM_DNN, a DNN network based on the self-attention mechanism. It is a deep learning model that utilizes the quadratic growth attention mechanism to improve performance and handle complex tasks. The model consists of an input layer, four hidden layers, an attention mechanism layer and an output layer. The front two hidden layers have 8 neurons each, while the last two hidden layers have 16 neurons each. ReLU is used as the activation function for all hidden layers. The attention mechanism layer is located after the hidden layers, making it easier to adjust inputs and weights. The scale and complexity of the KCCAM_DNN model can be better understood as shown in Table 2 of the model parameters. A visual representation of the model is provided in Fig 3.

**Table 2:** Parameter table.

Model	Input Layer Neurons	Hidden Layer1 Neurons	Hidden Layer2 Neurons	Hidden Layer3 Neurons	Hidden Layer4 Neurons	Attention Layer	Output Layer Neurons
DNN	4	8	8	16	16	-	3
**KCCAM_DNN**	2	8	8	16	16	1	3

**Fig 4:**
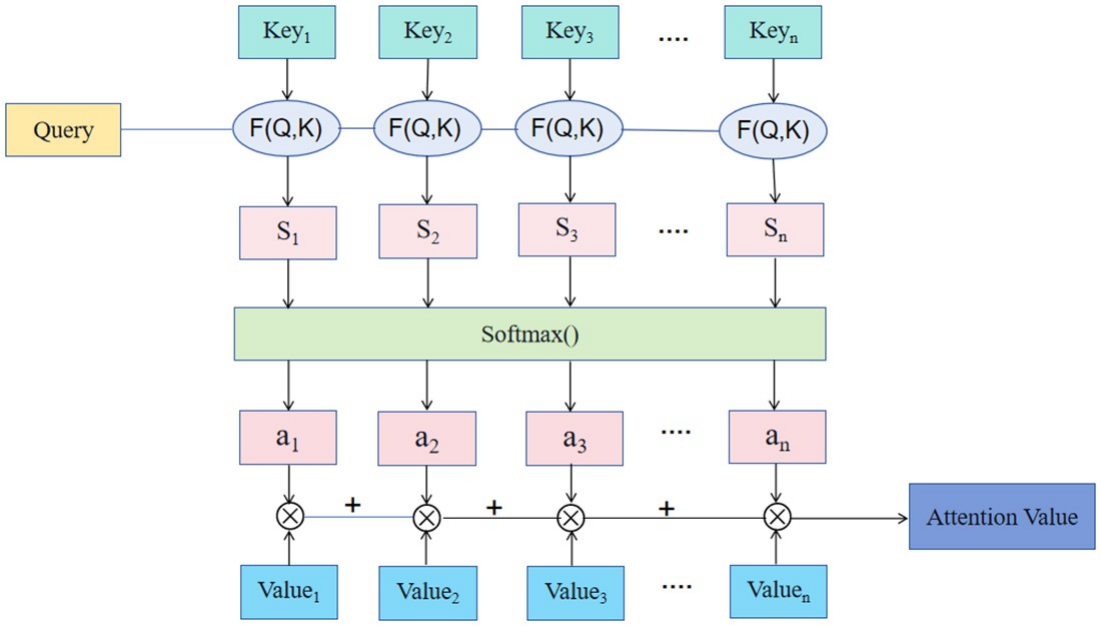
Flow chart of the self-attention mechanism.

The self-attention mechanism involves input mapping and the calculation of attention weights, as illustrated in Fig 4. Initially, the input features undergo mapping to the hidden space through a fully connected layer. Subsequently, attention scores are calculated and normalized. Finally, the input features are multiplied by the attention weights to obtain a weighted representation. The computation can be formalized as follows:

$$\begin{array}{c} Attention\left(Query,Key\right)=softmax(W_{a}*tanh\left(W_{q}*Query+W_{k}*Key\right)) \end{array}$$ (3)

where *Query* represents the query vector, *Key* represents the key vector, *W*_*q*_, *W*_*k*_, and *W*_*a*_ denote the learnable weight matrices, *tanh* signifies the hyperbolic tangent activation function, and *softmax* denotes the normalization operation of the attention scores.

#### Parameter design

In order to enhance adaptability to data characteristics and improve overall model performance, we conducted optimization of model parameters with the aim of maximizing key performance indicators.

Table 3 provides a concise overview of the hyperparameters utilized by the three classifiers: RF, SVM, and KCCAM_DNN. To achieve the optimal model configuration, we employed a grid search methodology for optimizing the model parameters. This systematic and detailed search process involves testing various combinations of parameters to identify the most suitable model configuration. Model performance is assessed by iterating through all parameter combinations in the space and conducting k-fold cross-validation [25] multiple times for each combination. The synergy of repeated cross-validation and grid search aids in discovering the most effective combination of hyperparameter settings for a defined parameter set [26], ultimately enhancing the predictive performance of the model.

**Table 3:** Experimental parameter design.

Items	Parameters	Description	Default	Optimization
RF	Max-Depth	Controls how specialized each tree is to the training dataset. The more the value the more likely overfitting.	2	3
	Max-Features	The maximum allowable number of trees the RF will consider for each split.	3	2
	n-Estimators	The number of trees you want the algorithm to build.	50	10
SVM	C	A regularization parameter that controls the error of the misclassification of SVC to data.	100	1000
	Kernel	TA non-linear transformation function to map data to a high-dimensional space.	Rbf	Rbf
	Gamma	A nonlinear parameter that represents the separation line or decision region between classes.	0.0001	0.001
	Optimizer	An algorithm that minimizes the loss function of the network during training.	Adam	RMSProb
2GDNN	Epoch	Defines the number of passes made to the entire training dataset during training.	100	200
	Batch-size	The number of samples utilized in one iteration.	1	5
KCCAM_DNN	Epoch	Defines the number of passes made to the entire training dataset during training.	100	200
	Batch-size	The number of samples utilized in one iteration.	1	5
	Learning rate	The size of the step is an important factor in the gradient descent optimization process.	0.001	0.001
	momentum	The technique can be used to accelerate gradient descent algorithms and help escape local minima.	0.9	0.9
K-Fold	n-Splits	The number of different validations set to create from the given train data.	10	10
	n-repeats	The Number of times cross-validation is repeated.	-	3
PIMA	Train	Percentage of the dataset for training.	582	582
	Test	Percentage of the dataset for testing.	146	146

### Model interpretation

Most contemporary deep models are intricate black-box models, posing challenges in interpreting their internal mechanisms and limiting their clinical applicability. To address this, we incorporated SHAP into the KCCAM_DNN model to elucidate and analyze the factors influencing diabetes within the model, thereby enhancing its interpretability.

SHAP (Shapley Additive exPlanations) is an open-source tool and method designed for interpreting machine learning model predictions. In 2017, Lee et al. [27] first proposed SHAP as a widely applicable method for explaining diverse models, particularly black-box models that are challenging to comprehend. Grounded in game theory’s Shapley value concept, SHAP investigates the contribution of the extracted features by visualizing the high-contributing features from the entire feature set using machine learning algorithms [28]. Shahid Akbar et al. [29] employed a global interpolation method utilizing SHAP to reduce the dimensionality of training vectors by selecting an optimal feature set, focusing on high-contributing features for target classification. The selected feature set was then trained using Bi-directional Temporal Convolutional Network (BiTCN). The resulting iAFPs-Mv-BiTCN model achieved an accuracy of 98.15% on the training dataset, demonstrating the effectiveness of their approach. The combination of SHAP and KCCAM_DNN model enhances interpretability by offering detailed insights into the importance of features, interactions, model understanding and identification of key risk factors associated with diabetes prediction. This knowledge is invaluable for developing more effective predictive models and guiding clinical decision-making processes for diabetes management. The model’s prediction value is the sum of the prediction mean of all samples and the SHAP value of each feature, expressed by the formula:

$$\begin{array}{c} Y=f_{b}+f_{1}+\ldots +f_{i}+\ldots +f_{M} \end{array}$$ (4)

where *Y* is the model’s predicted value, *f*_*i*_ is the SHAP value corresponding to feature *i*, and *f*_*b*_ is the mean of the predicted values of all samples.

By accurately calculating SHAP values that reflect the impact of each feature on the model, we enhance interpretability, providing a comprehensive understanding of the influence of each feature variable on diabetes prediction in diabetes research.

## Results and discussion

We employed various experimental settings to assess the performance of KCCAM_DNN in predicting diabetes, encompassing the following aspects: (1) assessment of the data preprocessing methods, (2) performance evaluation of the KCCAM_DNN model, (3) computation efficiency of KCCAM_DNN, and (4) model interpretation and analysis based on SHAP.

### Dataset description

We conducted experiments on the PIMA Indian and LMCH diabetes datasets.

The PIMA Indian diabetes dataset, sourced from the National Institute of Diabetes, Digestive and Kidney Diseases (NIDDK), comprises 768 cases involving Pima Indian females aged 21 years or older [30]. It includes 268 patients in the diabetes category and 500 in the non-diabetes category. Each data point has 8 characteristic features and 1 categorical label: pregnancies, glucose level, blood pressure, skinfold thickness, insulin level, body mass index (BMI), diabetes pedigree function, and age. Table 4 provides a description of the PIMA Indian dataset, with the outcome denoting the category label of each data item (0 for not having diabetes and 1 for having diabetes).

**Table 4:** Description of the PIMA Indian diabetes dataset.

S/N	Feature	Description	Missing Value
1	Pregnancies	Number of pregnancies	110
2	Glucose	Glucose concentration (2h oral test)	5
3	Blood Pressure (BP)	Diastolic blood pressure	35
4	Skin Thickness (ST)	Skin fold thickness in mm	227
5	Insulin	2h insulin serum (mm u/ml)	374
6	BMI	Body mass index = weight in kg/height in *m*^2^	11
7	Diabetes Pedigree Function (DPF)	Likelihood value computed from the relationship between the patient and the genetic history of the patient’s relative	0
8	Age	Age in years	0

The LMCH dataset is a diabetes dataset from the Laboratory of Medical City Hospital (LMCH), consisting of data from 1000 Iraqi patients [31]. It includes 103 with normal glucose levels, 53 with prediabetes, and 844 with diabetes.

### Evaluating indicator

We employ Precision, F1-score, Specificity, Recall, and Accuracy metrics to evaluate the predictive performance of the model in different configurations.

Typically, the positive class is designated as the class of interest, while the other classes are considered the negative class. The classifier’s predictions on the test dataset and their accuracy can be represented through a confusion matrix, as shown in Table 5.

**Table 5:** Confusion matrix for diabetic prediction evaluation. TP: model correctly predicts positive as positive. FN: model wrongly predicts positive as negative. FP: model wrongly predicts negative as positive. TN: model correctly predicts negative as negative.

	Predicted Positive(Diabetes)	Predicted Negative(No Diabetes)
Actual Positive(Diabetes)	True Positive (TP)	False Negative(FN)
Actual Negative(No Diabetes)	False Positive(FP)	True Negative(TN)

Precision measures the accuracy of correctly identifying diabetes in patients, calculated as the ratio of TP to the total of TP and FP. The formula is:

$$\begin{array}{c} Precision=\frac{TP}{TP+FP} \end{array}$$ (5)

Specificity is the proportion of negative instances accurately identified as nondiabetic. It is calculated as the TN divided by the sum of TN and FP. The formula is as follows:

$$\begin{array}{c} Specificity=\frac{TN}{TN+FP} \end{array}$$ (6)

Recall is the percentage of positive cases correctly identified as diabetic, calculated as TP divided by the sum of TP and FN. The formula is as follows:

$$\begin{array}{c} Recall=\frac{TP}{TP+FN} \end{array}$$ (7)

F1-score combines precision and recall, weighted by their influence on the overall outcome, accounting for both false positives and negatives. The formula is as follows:

$$\begin{array}{c} F1-score=2*\frac{Precision*Recall}{Precision+Recall} \end{array}$$ (8)

Accuracy denotes the proportion of accurate predictions over the total number of predictions, and can be expressed as follows.

$$\begin{array}{c} Accuracy=\frac{TP+TN}{TP+FP+TN+FN} \end{array}$$ (9)

### Performance evaluation of the data preprocessing methods

The data preprocessing stage involved the use of feature selection methods to eliminate irrelevant features and enhance the efficiency of the datasets. Additionally, missing value interpolation methods were employed to supplement data completeness.

The feature selection performance was evaluated using the LMCH dataset, which included prediabetes types and did not contain missing values. Table 6 displays the results, indicating that feature selection boosts classifier performance by 1.334%, 7.5%, and 0.915% for the SVM, RF, and KCCAM_DNN models, respectively. This outcome demonstrates that after feature selection, the performance of the RF models is strengthened. Henceforth, all analyses employ a subset of the dataset post-feature selection as input.

**Table 6:** Performance evaluation of feature selection. Where, FS stands for feature selection.

Set	Model	Precision(%)	Recall(%)	F1-Socre(%)	Train Acc(%)	Test Acc(%)
No FS	SVM	94.385	94.000	93.714	95.429	94.000
	RF	88.651	92.500	90.432	92.000	92.500
	KCCAM_DNN	96.074	96.000	95.911	100	95.999
With FS	SVM	96.551	93.768	94.912	98.545	94.915
	RF	100	100	100	98.726	100
	**KCCAM_DNN**	**97.522**	**97.333**	**97.393**	**97.424**	**97.333**

**Fig 5:**
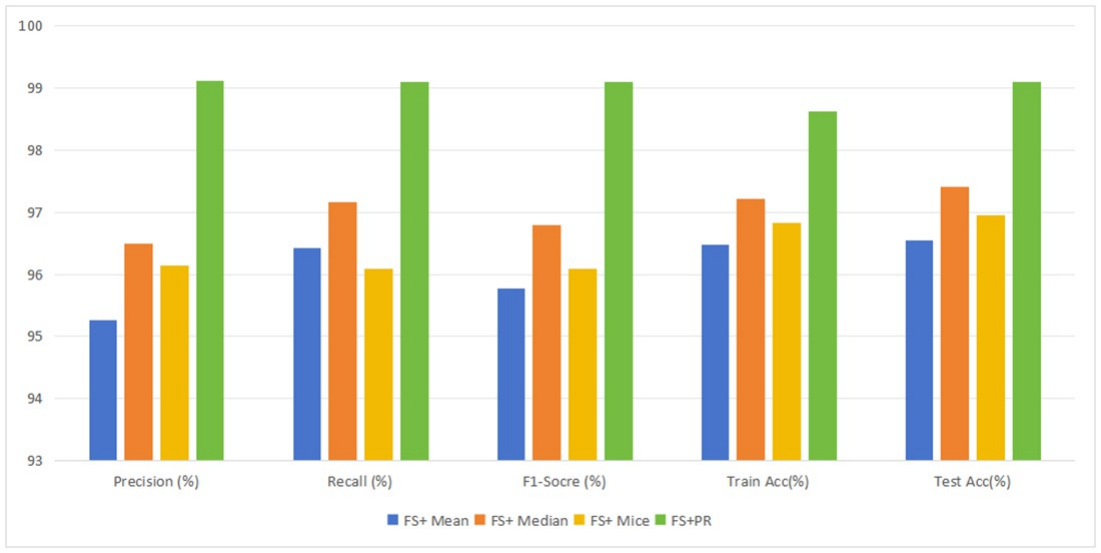
Evaluation of missing value imputation methods.

Next, we evaluate the missing value interpolation method using the PIMA Indian dataset. The performance of the mean, median, and MICE missing value interpolation methods was compared to the PR method. As shown in Table 7 and Fig 5, the PR predictive interpolation method outperforms common methods, indicating its efficacy in resolving missing values in nonlinearly distributed data. Multiple Imputation Chained Equations (MICE) does not perform as well as polynomial regression (PR) in terms of accuracy, with a 2.134% difference in performance.

**Table 7:** Performance evaluation of the missing value imputation methods. Where, FS stands for feature selection, PR stands for polynomial regression.

Data Preprocessor	Precision(%)	Recall(%)	F1-Socre(%)	Train Acc(%)	Test Acc(%)
FS+ Mean	95.259	96.421	95.772	96.480	96.551
FS+ Median	96.497	97.161	96.796	97.206	97.413
FS+ Mice	96.139	96.084	96.082	96.828	96.956
**FS+PR**	**99.109**	**99.090**	**99.089**	**98.624**	**99.090**

**Fig 6:**
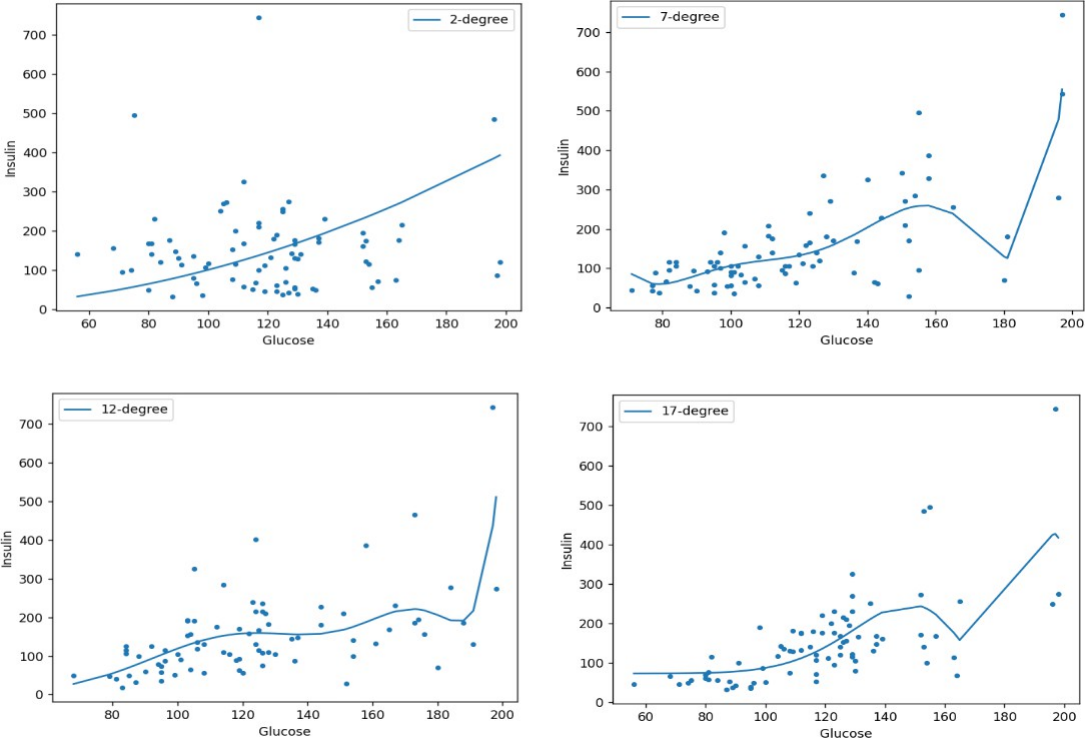
The fit of the polynomial regression line for the glucose predictor to the predicted values of insulin is depicted in Fig 6. Various nth-degree polynomials, including 2(Upper left), 7(Upper right), 12(Lower left), and 17(Lower right), are plotted. The performance summary indicates that a 7-degree polynomial provides a better fit.

The predictive capability of PR relies on evaluating the nth-order polynomial to determine the optimal fit for insulin data. Analysis (Fig 6 and Table 8) with the Root Mean Square Error (RMSE) and R-squared (*R*^2^) error indicates that the 7th-order polynomial aligns more accurately with the data.

**Table 8:** Evaluation of nth order polynomials with RMSE and *R*^2^.

N-Degree	RMSE	*R* ^2^
2	82.90	**0.4391**
7	**76.38**	0.6081
12	80.65	0.4692
17	80.51	0.4710

### Performance evaluation of the KCCAM_DNN model

**Fig 7:**
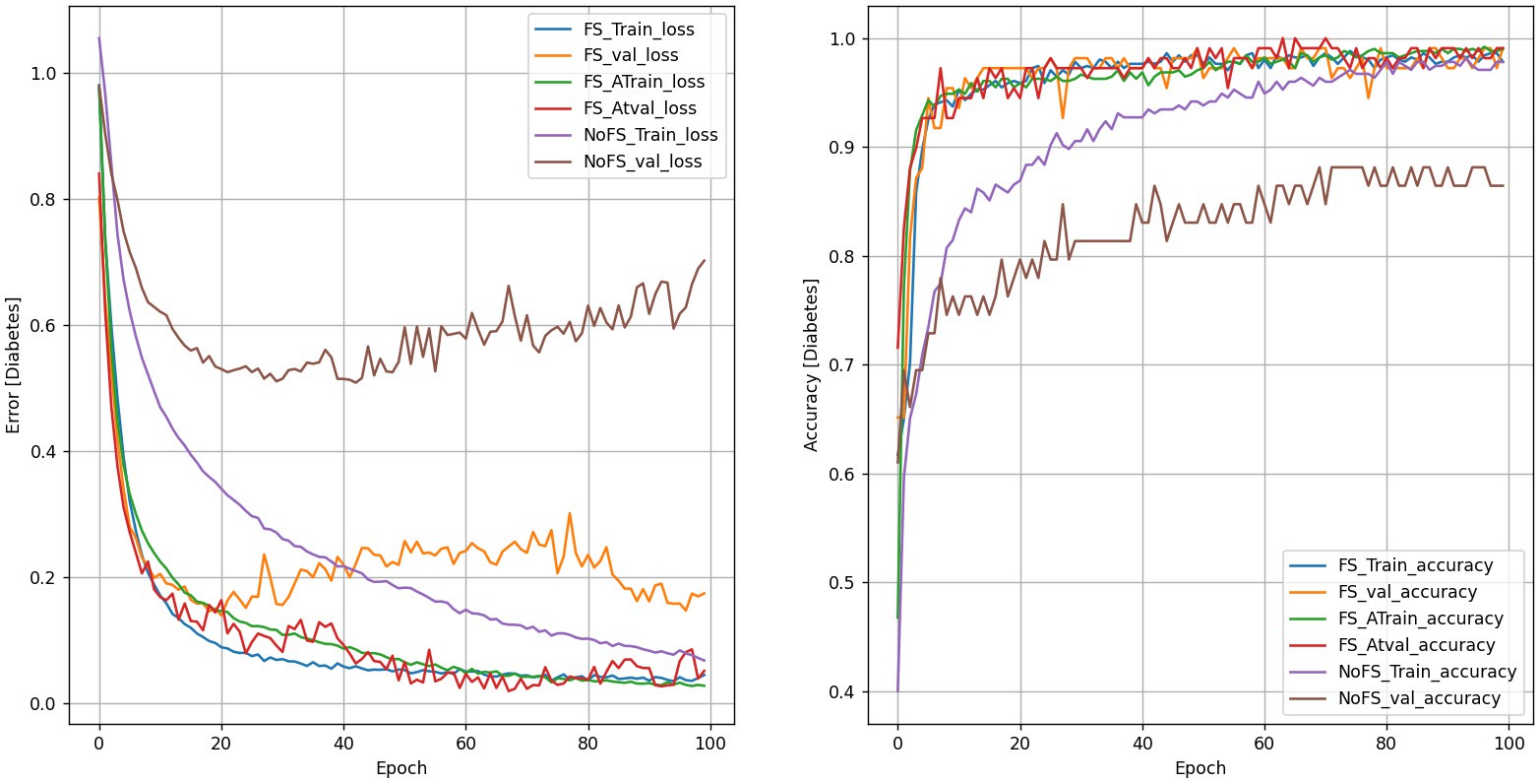
KCCAM_DNN performance on PIMA Indian diabetes dataset.

**Fig 8:**
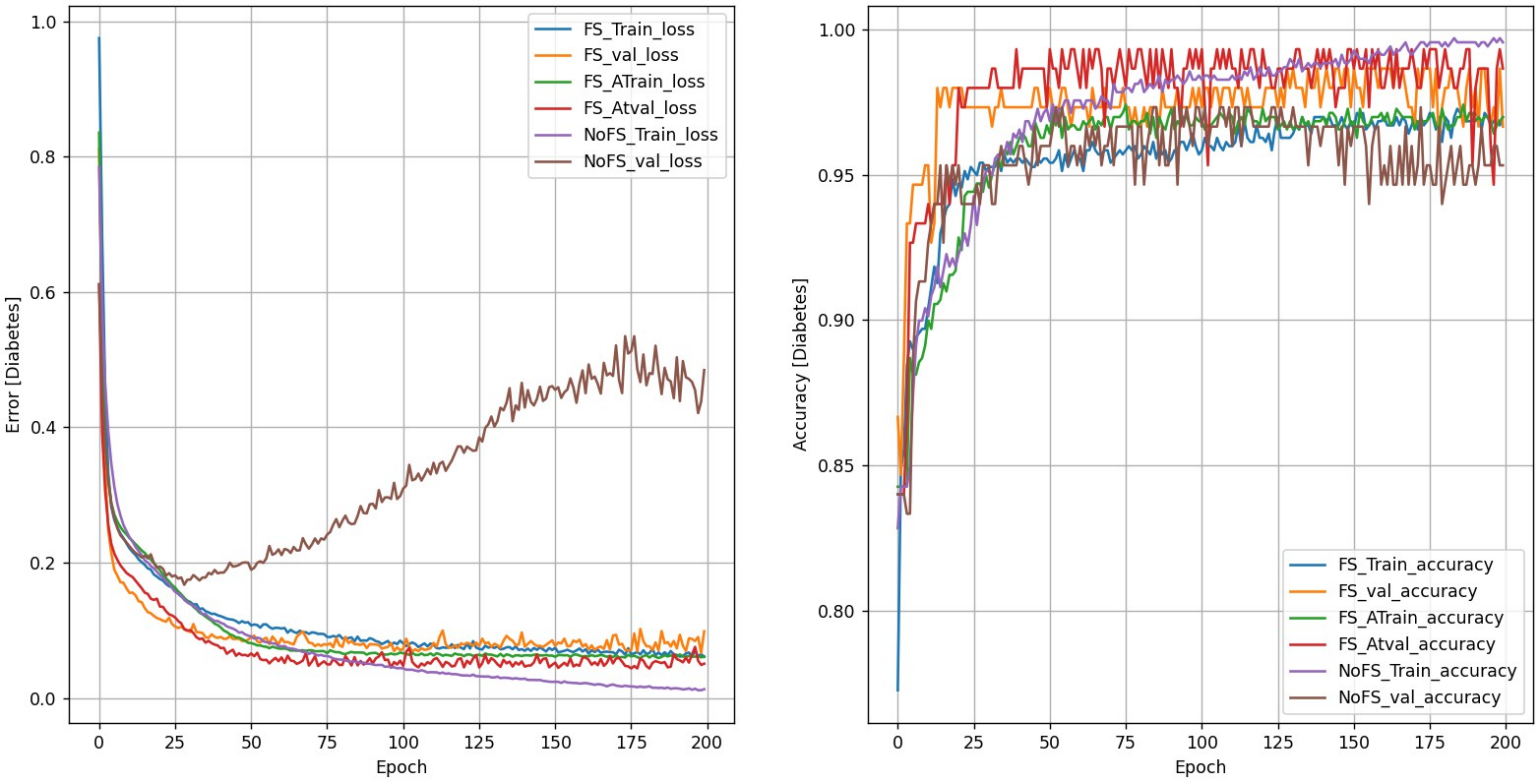
KCCAM_DNN performance on Laboratory of Medical City Hospital dataset.

We compared KCCAM_DNN against some commonly used machine learning methods and deep models. The overall benchmark results are shown in Figs 7 and 8 and Table 9. The test accuracy reaches 99.090% and 99.333%. Comparing with the best available methods [15], the test accuracy is improved by 1.842% and 2% on PIMA Indian and LMCH datasets, respectively. As can be observed in Table 9, ORF shows a higher probability of determining the severity of the diagnosis at 100% than KCCAM_DNN. However, in a broader context, KCCAM_DNN can be better used when the number of datasets is large, in which case ORF may fail because it is only stable with a small amount of data [32].

**Table 9:** Performance evaluation of diabetes prediction. Where, FS stands for feature selection, MVI stands for missing value imputation, O* stands for parameter optimisation.

Data	Data Preprocessing	Model (%)	Precision (%)	Recall (%)	F1-Socre (%)	TrainAcc (%)	TestAcc (%)
LMCH	FS	SVM	96.551	93.768	94.912	98.545	94.915
		OSVM	97.674	95.333	95.299	97.281	95.333
		RF	100	100	100	98.726	100
		ORF	98.765	95.238	96.969	98.247	95.000
		2GDNN	97.348	96.667	96.965	98.714	96.667
		O2GDNN	97.281	97.333	97.265	99.571	97.333
		**KCCAM_DNN**	99.362	**99.333**	99.339	97.424	**99.333**
		**OKCCAM_DNN**	**100**	98.850	**99.421**	**97.622**	98.000
PIMA	FS+MVI	SVM	97.083	96.363	96.375	99.607	96.363
		OSVM	98.462	98.462	98.462	100	98.181
		RF	100	100	100	98.109	100
		ORF	**100**	**100**	**100**	**100**	**100**
		2GDNN	95.156	94.495	94.504	99.802	94.495
		O2GDNN	97.348	97.245	97.255	99.012	97.248
		**KCCAM_DNN**	99.109	99.090	99.089	99.803	99.090
		**OKCCAM_DNN**	98.989	98.666	98.806	99.312	98.630

### Computation efficiency of KCCAM_DNN

KCCAM_DNN significantly enhances temporal performance, with a training time of 64.049 seconds. Table 10 shows its superior performance over comparison models. Compared with the best available methods [15], the time improvement is 17.941 seconds.

**Table 10:** Time evaluation of the proposed KCCAM_DNN.

Model	Precision(%)	Recall(%)	F1-Socre(%)	TrainAcc(%)	TestAcc(%)	Time(s)
2GDNN	95.156	94.495	94.504	99.802	94.495	81.990
**KCCAM_DNN**	**99.109**	**99.090**	**99.089**	**99.803**	**99.090**	**64.049**

The experiments were conducted on a system with the Windows 11 operating system. Our method does not require dedicated GPU devices and performs efficiently using only CPU devices. The machine is powered by an Intel(R) Core(TM) i7-8550U CPU processor with a base frequency of 1.80GHz, a maximum turbo frequency of 2001 MHz, 4 cores, and 8 logical processors. The system is configured with 81GB of RAM. The development language used for the experiments was Python 3.9.1. KCCAM_DNN exhibits outstanding performance in managing large datasets and providing swift response times for individual predictions, rendering it a high-quality model for real-time applications. This creates the opportunity for its utilization in actual clinical scenarios.

### Model interpretation and analysis based on SHAP

SHAP can effectively analyze the key factors that affect diabetes prediction, significantly improving model transparency and reliability. This insight plays a crucial role in gaining a deeper understanding of the pathogenesis of diabetes and developing targeted prevention strategies.

**Fig 9:**
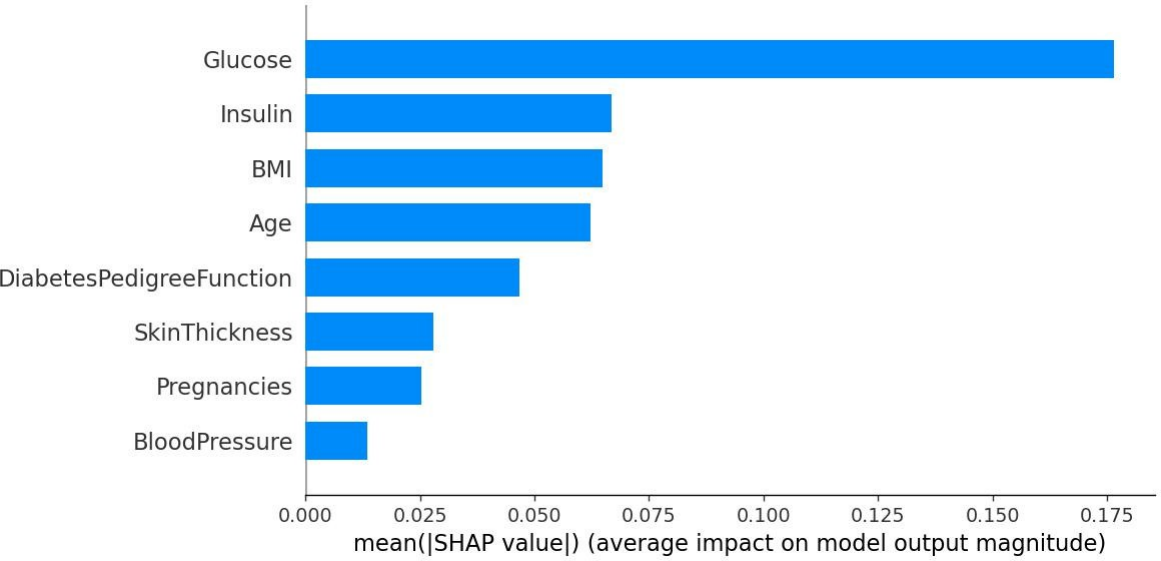
An analysis of the importance of influencing the characteristics of diabetes.

Fig 9 displays SHAP bars organized by the corresponding SHAP value of each feature, indicating the significance of global characteristics. As shown in Fig 9, characteristics such as glucose concentration and insulin have a noteworthy influence on the classification of diabetes, reaching SHAP values of 0.182 and 0.067 respectively, with discrepancies in glucose concentration yielding the most notable impact on the model. This finding is highly consistent with the key features identified after using Kendall’s correlation coefficient for feature selection, both focusing on the two core features of glucose and insulin. After feature selection, the model’s prediction accuracy has improved, achieving a 0.915% increase in performance compared to the data without feature selection, ultimately reaching an accuracy of 97.333%. This fully validates the importance of feature selection in improving model performance.

**Fig 10:**
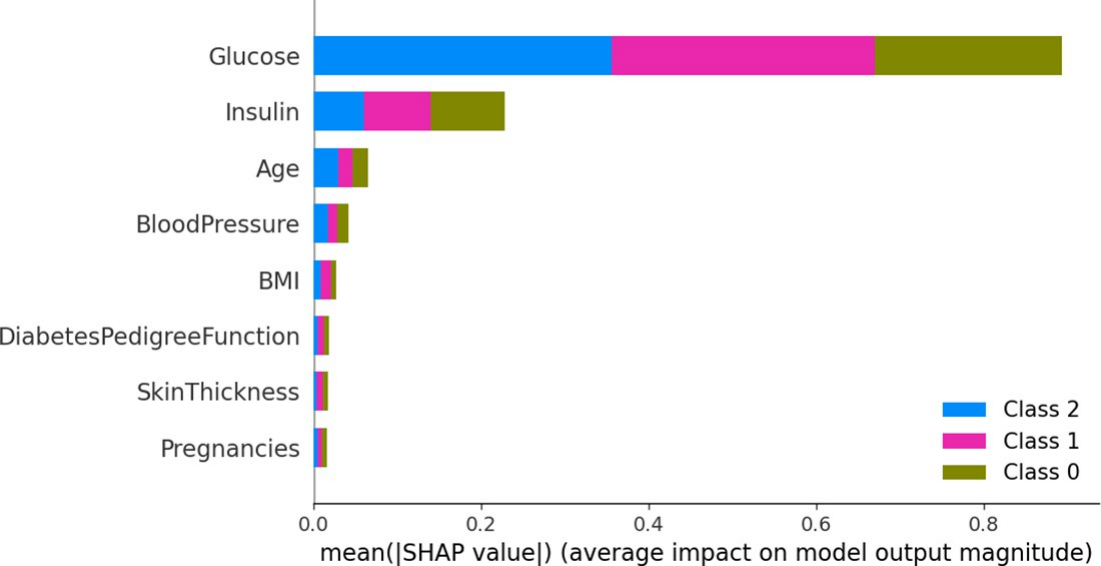
Average impact on model output magnitude.

Fig 10 shows the average impact of each feature on the model output in the diabetes datasets. It provides information about the average relative importance of features concerning the model predictions. The results demonstrate that the average impact of glucose concentration and insulin on the model is significant, further validating their importance in diabetes prediction.

**Fig 11:**
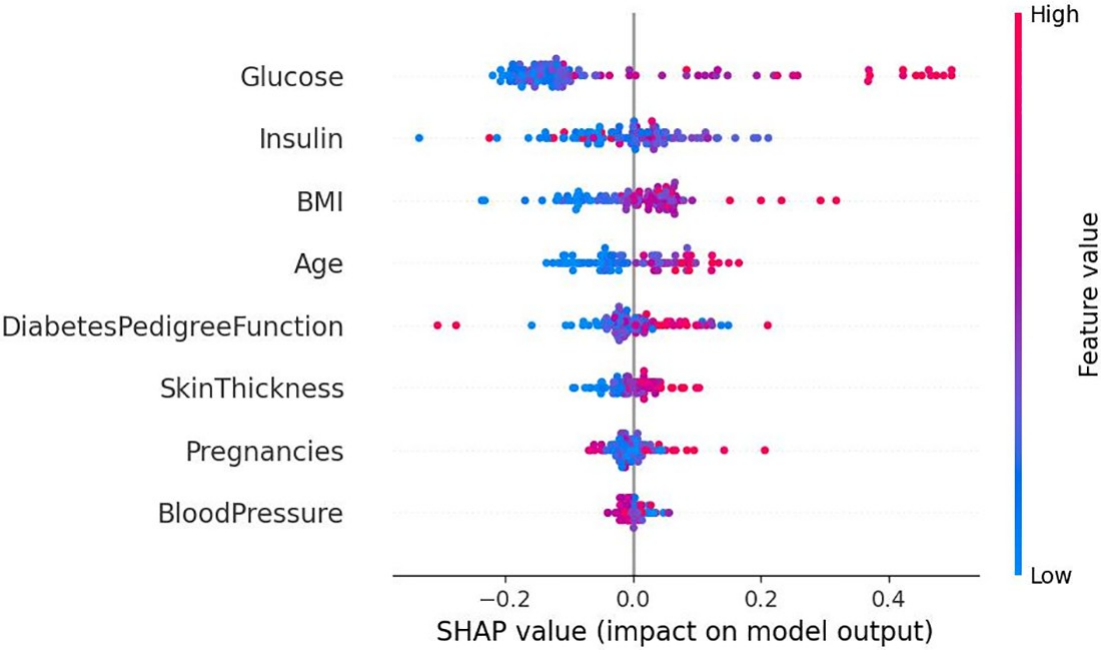
SHAP feature analysis summary chart.

Fig 11 displays the SHAP summary plot, ranking the significance of features that influence determining diabetes. The graph visually presents the distribution of SHAP values for each feature, with the horizontal axis reflecting the magnitude of the SHAP values, and the vertical axis arranging the samples based on the sum of SHAP values for that feature. Each data point represents a single sample, with the sample size stacked vertically. The color indicates the level of the feature value, with red indicating a high value and blue indicating a low value, and a wider color region signifies a more significant impact of that feature on the model’s predictions. As shown, both glucose and insulin characteristics have a significant impact on predicting diabetes status. As the values of these characteristics decrease, the likelihood of being diagnosed with diabetes decreases. Of these, glucose concentration has the most significant effect on the identification of diabetes. Therefore, we need to control glucose and insulin effectively afterward to better prevent diabetes.

By analyzing the above three figures, we can clearly observe that the analysis results of the SHAP model and the conclusions from feature selection are highly compatible in terms of key feature identification. The mutual validation of these two methods enables us to understand more accurately the influence of features on diabetes prediction, providing a strong basis for subsequent model optimization and decision support.

## Conclusion

In conclusion, we present KCCAM_DNN, a high-performance model designed for predicting and diagnosing diabetes. KCCAM_DNN leverages Kendall’s correlation coefficients for feature selection, which accelerates model training and inference, and demonstrates proficiency in handling nonlinear data. Polynomial regression is used to ensure robust missing value interpolation, preserving data integrity. The proposed deep neural network, KCCAM_DNN, incorporates a self-attention mechanism to prioritize crucial feature information impacting diabetes, enhancing prediction performance. The SHAP model contributes to accurately understand the impact of each feature on the model, facilitating clinical application. Experimental results indicates the superior performance of KCCAM_DNN in diabetes prediction compared to current state-of-the-art methods. Future efforts will focus on optimizing feature analysis and hyperparameter settings, in order to further enhance model accuracy and extend its application to real-world scenarios to provide more accurate prediction services for diabetes patients. By collecting patients’ blood glucose monitoring data and combining them with lifestyle habits such as diet, exercise and sleep, we can predict whether people are at risk of developing diabetes and help doctors design more effective prevention and treatment strategies.
